# Acute Effect of the Timing of Resistance Exercise and Nutrient Intake on Muscle Protein Breakdown

**DOI:** 10.3390/nu12041177

**Published:** 2020-04-22

**Authors:** Wataru Kume, Jun Yasuda, Takeshi Hashimoto

**Affiliations:** Faculty of Sport and Health Science, Ritsumeikan University, 1-1-1 Nojihigashi, Kusatsu, Shiga 525-8577, Japan; w.kume0913@gmail.com (W.K.); 55fhyanh@gmail.com (J.Y.)

**Keywords:** muscle protein breakdown, resistance exercise, meal timing, protein, carbohydrate, 3-methyhistidine, insulin, crossover-design study, acute response

## Abstract

Background: Combining resistance exercise (RE) with nutrient intake stimulates muscle protein net balance. However, it is still unclear whether the optimal timing of nutrient intake is before or after RE, especially on muscle protein breakdown (MPB) for an augmented muscle anabolic response. The aim of this study was to investigate the effect of a substantial mixed meal (i.e., nutrient- and protein-dense whole foods) before or after RE, compared with RE without a meal on the acute response of MPB in a crossover-design study. Methods: Eight healthy young men performed three trials: (1) meal intake before RE (Pre), (2) meal intake after RE (Post), and (3) RE without meal intake (No). Plasma insulin and 3-methylhistidine (3-MH), an MPB marker, were measured. Results: Time course change in plasma insulin level after RE was significantly higher in the Post condition than in the Pre and No conditions. The area under the curve of 3-MH concentration was significantly lower in the Post condition than in the Pre and No conditions. Conclusions: These results suggest that a substantial mixed meal immediately after RE may effectively suppress MPB in the morning.

## 1. Introduction

Resistance exercise (RE) maintains and increases muscle mass by inducing an anabolic response in which muscle protein synthesis (MPS) surpasses muscle protein breakdown (MPB) [1]. However, performing RE in a fasted state promotes not only MPS but also MPB after RE by increased proteasome activity [2], resulting in an attenuated muscle anabolic response. In this regard (i.e., the promotion of MPB in a fasted state), it is important to provide nutrient intake to upregulate muscle protein net balance (MPS minus MPB) [2,3,4,5]. For instance, the suppression of MPB following RE may contribute to upregulated muscle protein net balance and thus increased muscle mass [2,3,6,7].

In addition, the timing of nutrient intake is also important for the anabolic response of RE. Rasmussen et al. (2000) reported that ingestion of amino acid (AA) and carbohydrate (CHO) at 1 h post-exercise tends to suppress MPB compared with the effect of ingestion at 3 h post-exercise [8]. This indicates that even if a substantial amount of nutrients is consumed after RE, the timing of nutrient intake is important for the suppression of MPB. However, it remains unclear whether nutrient intake before or after RE is optimal to maximize the anabolic response [9]. In a previous study that examined the timing of substantial nutrient intake in relation to acute RE, Tipton et al. (2001) reported that AA and CHO intake immediately before RE promoted the response of MPS compared to that immediately after RE [10]. On the other hand, Fujita et al. (2009) suggested that AA and CHO intake an hour before RE did not enhance the MPS response after RE compared to RE without any nutrient intake [11]. Given that AA and CHO intake after RE promoted MPS compared to the effect of RE without any nutrient intake [12], Fujita et al. suggested that nutrient intake after RE may promote MPS to a greater extent than nutrient intake before RE [11]. While nutrient timing might be relevant to increase MPS or decrease MPB, the long-term effects are debatable. Recently, in a longitudinal study, whey protein supplementation consumed either pre- or post-resistance training similarly promoted skeletal muscle mass, muscular strength, and functional capacity in pre-conditioned older women [13]. In another longitudinal study, the timing of protein consumption had no effect on lean mass, muscular strength, or functional capacity combined with resistance training in postmenopausal women [14]. Moreover, few studies examined the timing effects of nutrients on MPB, even though anabolic response should be in consideration with both MPS and MPB [2]. Importantly, the small number of studies limits the ability to draw a firm conclusion on the effects of ingesting nutrient- and protein-dense whole foods on the regulation of postprandial protein metabolism, although nutrient-dense whole foods, compared with supplemental isolated protein sources, as mostly investigated in the above studies, are more commonly consumed within a normal eating pattern to achieve daily protein recommendations and concomitant improvements in diet quality [15,16,17].

It is widely thought that breakfast is the most important meal of the day for maintaining human health [18]. However, up to 35% of young people in many Western countries regularly skip breakfast [19], and 30.6% of men and 23.6% of women in Japan do so as well [20]. Of note, in the morning, negative muscle protein net balance is brought about by a long period of fasting after the last meal [21]. Yasuda et al. reported in a cross-sectional study that having breakfast with a high amount of protein in addition to CHO leads to maintenance and/or enhancement of muscle mass [22,23], which is important for health improvement. Enhancement of muscle mass helps in the prevention of metabolic syndromes [24], diabetes [25], and sarcopenia [26]. In addition to minimizing muscle loss in older people, maximizing muscle in youth and young adulthood and maintaining muscle in middle age are warranted to prevent or delay sarcopenia [27]. These findings can be interpreted to indicate that ingesting a substantial meal (i.e., nutrient- and protein-dense whole foods) at breakfast is a pivotal event in daily life to achieve positive muscle protein net balance, and hence human health, even in young people.

Based on these backgrounds (i.e., inconsistency regarding optimal nutritional timing for anabolic response from RE and importance of a substantial meal at breakfast for human health through the maintenance of muscle mass), further information on the response of MPB to exercise and nutrition would advance our understanding of muscle metabolism and exercise, as well as the influence of exercise variables and nutrition on training adaptations. This information may be useful not only to athletes, but also to better understand overall metabolic health for all people [2]. The aim of this study was to examine the effect of the timing of the intake of a substantial breakfast (i.e., mixed meal) before or after RE on the acute response of MPB compared with the effect of RE without nutrient intake in a crossover-design study. Given that postprandial hyperinsulinemia inhibits increased MPB after RE [5,28], we hypothesized that a mixed meal after RE may suppress MPB, promoting anabolic response, compared to the effect of a mixed meal before RE or RE without a meal. 

## 2. Materials and Methods 

### 2.1. Participants

Eight healthy young men (age: 23.3 ± 0.3 years old, height: 171.5 ± 1.8 cm, weight: 63.6 ± 1.5 kg, body fat percentage: 17.4 ± 1.8%, lean mass: 52.6 ± 1.3 kg, BMI: 21.6 ± 0.4) were recruited. Prior to the experiment, screening was conducted based on questionnaires used to collect information on participants’ characteristics, such as allergies, medical history, exercise, smoking, and alcohol consumption habits. This study was approved by the Ethics Committee for Human Experiments at Ritsumeikan University (BKC-IRB-2019-004) and was conducted in accordance with the Declaration of Helsinki. Written informed consent was obtained from all participants.

### 2.2. Experimental Design

**Figure 1:**
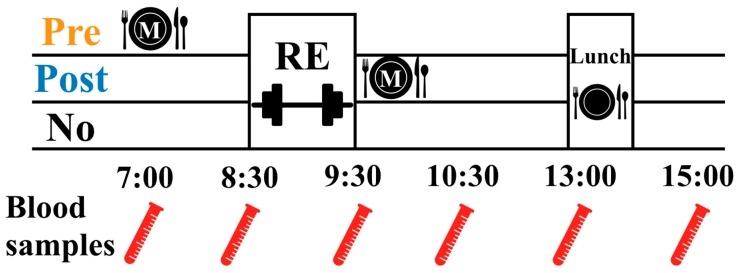
Experimental protocol. RE, resistance exercise; M, meal; Pre, meal intake before RE; Post, meal intake after RE; No, RE without meal intake.

In this study with a crossover design in a randomized and counterbalanced order, all participants underwent three conditions: meal intake before RE (Pre), meal intake after RE (Post), and RE without meal intake (No). The participants refrained from intense exercise, intake of animal protein, caffeine, and alcohol for three days before each experimental day and maintained normal nighttime sleep durations (a minimum of approximately eight hours). The experiment was conducted for eight hours from 7:00 to 15:00. RE was performed for an hour from 8:30 to 9:30 in all conditions. The timing of the meal intake was different for each condition. The meal time of the Pre condition was 7:00–7:15, before RE, while that of the Post condition was 9:30–9:45, immediately after RE, and the No condition was characterized by no meal intake before or after RE. Lunch time was at 13:00–13:15 in all conditions. Biological blood samples were collected at 7:00, 8:30, 9:30, 10:30, 13:00, and 15:00 (Figure 1).

### 2.3. Pre-Measurement

Height, weight, body fat mass, and lean body mass (LBM) were measured by height and weight scales (TANITA Co., WB-510, Tokyo, Japan) and a biochemical electrical impedance device (InBody Japan Co., InBody 720, Tokyo, Japan). At a week prior to the experiment, one repetition maximum (1RM; the maximum weight that can be lifted for one repetition) was measured by a qualified trainer on the following weight-stack machines: chest press, seated row, shoulder press, leg extension, and seated leg curl (Life Fitness Japan Ltd., Tokyo, Japan).

### 2.4. Meal Control

The participants were instructed to follow a three-day meat-free diet including no animal protein (chicken, beef, pork, fish, seafood, etc.) to return blood concentrations of 3-MH to baseline levels [29]. Each participant abstained from food (overnight fasting) for 12 h before the experiment.

### 2.5. Meal Contents

The dietary content of the breakfast meal included a high protein level based on the findings that sufficient protein intake at breakfast [22] and daily protein intake of 1.62 g/kg (body weight) should be ensured for muscle hypertrophy [17]. Additionally, the energy intake at breakfast was set at 50% of the average daily calorie intake for young people [22], and the following menu was consumed for breakfast: soybean granola 100 g (Gorotto granola, Nisshin Cisco Inc., Osaka, Japan), Greek yogurt 100 g (Meiji THE GREEK YOGURT plain, Meiji Co., Tokyo, Japan), whey protein 32 g (MYPROTEIN, The Hut group, Cheshire, UK), milk 200 mL (Biwako milk, Japan Dairy Association, Osaka, Japan), and a banana (produced in the Philippines) (total energy, 868 kcal; protein, 55.4 g (25.6%); fat, 24.8 g (25.8%); carbohydrate, 105.6 g (48.6%)). For lunch, a slice of bread (Hyozyuku, Yamazaki Bread Co., Tokyo, Japan), a butter roll (Royal Bullet Butter Roll, Yamazaki Bread Co., Tokyo, Japan), milk 200 mL (Biwako milk, Japan Dairy Association, Osaka, Japan), strawberry jam 60 g (55 Strawberry, Aohata Co., Hiroshima, Japan), and canned orange 182 g (Kintaiyo Orange, Taiyo Food Co., Nagasaki, Japan) (total energy, 622 kcal; protein, 16.1 g (10.4%); fat, 13.7 g (19.8%); carbohydrate, 108.3 g (69.8%)) were provided.

### 2.6. Resistance Exercise

Under the supervision of a qualified trainer, the participants performed five types of REs (chest press, seated row, shoulder press, leg extension, and seated leg curl) at 70% 1RM intensity for an hour (8:30–9:30). The participants performed a warm-up (10 repetitions) at 50% 1RM intensity before each RE. A total of 10 repetitions × 3 sets were performed as the main session for each RE, with an interval time between sets of 2 min and a 3-min performance time for each RE. The speed of motion was 1 s contraction and 3 s extension. 

### 2.7. Blood Sampling

A winged needle was inserted into the forearm vein, and blood samples were collected at 7:00, 8:30, 9:30, 10:30, 13:00, and 15:00. After blood samples were gently inverted five times, the EDTA-treated blood collection tubes were then centrifuged for 10 min at 4 °C and 3000 rpm for plasma collection. The extracted plasma samples were stored frozen at −80 °C. Insulin and 3-MH concentrations were analyzed in the plasma by ELISA (Mercodia Human Insulin ELISA Kit, Mercodia Ltd., Uppsala, Sweden; 3-Methylhistidine ELISA Kit, Abbexa Ltd., Cambridge, UK). The ELISAs were run according to the manufacturer’s instructions, and a sample was run in duplicate. Optical density at 450 nm was obtained for the measurement of insulin and 3-MH. The intra-assay coefficients of variation were 5.6% and 7.6% for insulin and 3-MH, respectively. The inter-assay coefficients of variation were 7.9% for insulin and 9.2% for 3-MH.

### 2.8. Statistical Analysis

The data of the area under the curve (AUC) of the time course changes in plasma 3-MH concentration were analyzed using one-way analysis of variance (ANOVA). The AUC of plasma 3-MH concentration was calculated using data from 7:00 to 15:00. The time course changes in plasma insulin and 3-MH were analyzed using two-way (condition × time) repeated-measures ANOVA. Specific differences were identified with a Bonferroni post hoc test. The statistical significance level was defined as *p* < 0.05, and all values were the mean ± standard error. All statistical analyses were conducted with IBM SPSS software (Ver. 24, IBM Corp, Chicago, IL, USA).

## 3. Results

### 3.1. The Concentration of Plasma Insulin

**Figure 2:**
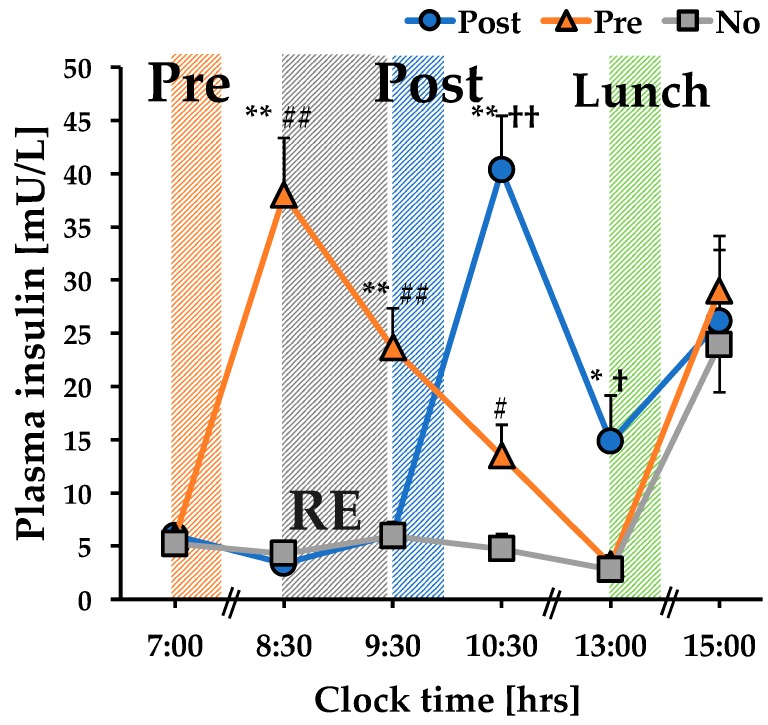
Time course change in plasma insulin. *; *p* = 0.043 Post vs. Pre, #; *p* = 0.023 Pre vs. No, †; *p* = 0.015 Post vs. No, **; *p* = 0.001 Post vs. Pre, ## *p* = 0.001 Pre vs. No, ††; *p* < 0.001 Post vs. No. RE: resistance exercise; Post: meal intake after RE; Pre: meal intake before RE; No: RE without meal intake.

The time course change in plasma insulin concentration showed interaction between condition and time (*p* < 0.001) (Figure 2). Plasma insulin concentrations in the Post condition at 10:30 and 13:00 were significantly higher than those in the Pre and No conditions (*p* = 0.001, *p* = 0.015, respectively). Additionally, the plasma insulin levels in the Pre condition at 8:30 and 9:30 were significantly higher than those in the Post and No conditions (*p* = 0.001), and at 10:30, the level in the Pre condition was significantly higher than that in the No condition (*p* = 0.023).

### 3.2. The Concentration of Plasma 3-MH

**Figure 3:**
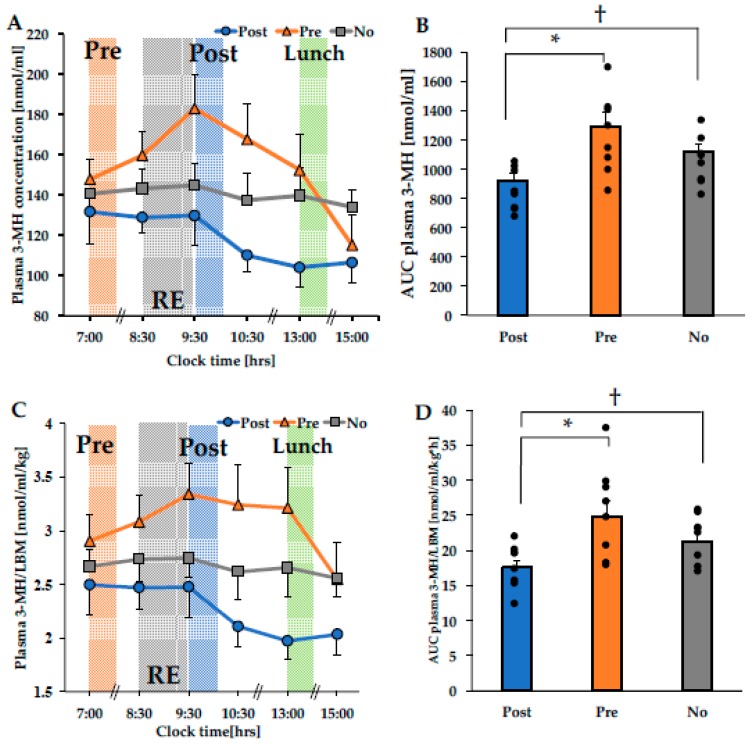
Time course change in plasma 3-MH concentration (**A**), the area under the curve of plasma 3-MH concentration (7:00–15:00) (**B**), time course change in plasma 3-MH/LBM levels (**C**), the area under the curve of plasma 3-MH/LBM levels (7:00–15:00) (**D**). * *p* = 0.016, † *p* = 0.007 (**B**), * *p* = 0.020, † *p* = 0.007 (**D**). RE: resistance exercise; Post: meal intake after RE; Pre: meal intake before RE; No: RE without meal intake; 3-MH: 3-methylhistidine; LBM: lean body mass.

The time course change in plasma 3-MH concentration indicated a main effect between conditions (*p* = 0.001), but there was no main effect or interaction between the time elements (Figure 3A). 

The AUC of plasma 3-MH concentration (7:00–15:00) showed a significant difference between conditions (*p* < 0.001) (Figure 3B). The AUC of the plasma 3-MH concentration in the Post condition was significantly lower than those in the Pre and No conditions (*p* = 0.016 Post vs. Pre, *p* = 0.007 Post vs. No). However, the AUC of the plasma 3-MH levels between Pre and No was not significantly different (*p* = 0.400).

Because the creatinine used for the correction of 3-MH was proportional to the LBM [30], 3-MH was corrected by LBM to calculate the amount of MPB per skeletal muscle mass. Thus, we calculated 3-MH/LBM levels as a main outcome in the present study. The time course change in plasma 3-MH/LBM levels indicated a main effect between conditions (*p* = 0.002), but there was no main effect or interaction between the time elements (Figure 3C). 

The AUC of plasma 3-MH/LBM levels (7:00–15:00) showed a significant difference between conditions (*p* = 0.010) (Figure 3D). The AUC of the plasma 3-MH/LBM levels in the Post condition was significantly lower than those in the Pre and No conditions (*p* = 0.020 Post vs. Pre, *p* = 0.007 Post vs. No). However, the AUC of the plasma 3-MH/LBM levels between Pre and No was not significantly different (*p* = 0.650).

## 4. Discussion

For the first time, the present study examined the MPB response to the timing of nutrient intake (after RE, before RE, or RE without nutrient intake) in a crossover-design study. Insulin secretion after RE increased in the order of Post and Pre conditions, confirming the difference in insulin kinetics in each condition (Figure 2). As a result, the Post condition suppressed 3-MH concentration after RE compared to the effects observed in the Pre and No conditions (Figure 3), while there was no difference in 3-MH secretion between the Pre and No conditions. Therefore, it was suggested that ingesting a substantial mixed meal immediately after RE was effective in suppressing MPB after RE and thus may enhance anabolic response (i.e., positive muscle protein net balance) in RE.

Insulin secretion after RE may be involved in the preventive effect of nutritional intake on MPB. It has been reported that the amount of insulin secreted as a result of nutrient intake required to suppress MPB is 30 mU/L [31]. As the combination of protein and CHO intake increases insulin levels additively [3,32], the combined intake of 55.4 g protein and 105.6 g CHO increased peak insulin level to approximately 40 mU/L after RE, which might have been sufficient to suppress MPB in the Post condition in the present study. On the other hand, the insulin level after RE in the Pre condition did not reach the required level (i.e., less than 25 mU/L), suggesting that the preventive effect of nutritional intake on MPB after RE was attenuated. In line with this finding, there was no difference in the MPB response the between Pre and No conditions in the present study (i.e., the plasma 3-MH and 3-MH/LBM levels between Pre and No were not significantly different (*p* = 0.400 and *p* = 0.650, respectively), which corresponds to the previous finding showing that essential AA and CHO intake either before RE or RE without nutrient intake similarly promoted MPS but reduced muscle protein net balance after RE by similarly promoting increased MPB after RE as well as MPS [11]. In addition, relatively low levels of post-lunch 3-MH concentration may be at least partially attributed to increased insulin secretion. However, we simply demonstrated coincidence between increased circulating insulin concentrations and 3-MH suppression, which might not elicit other MPB-related players (e.g. amino acids [28]) involved in decreased MPB. Further studies are needed to identify mechanistic evidence.

On the other hand, Tipton et al. (2001) suggested that essential AA and CHO intake immediately before RE promotes anabolic response to a greater extent than nutritional intake immediately after RE [10]. This discrepancy can be attributed to the timing of nutrient intake before RE. In the study by Tipton et al. [10] the nutritional intake occurred immediately before RE, while in the study by Fujita et al. [11] and the present study, nutritional intake occurred an hour or 1.5 h before RE, respectively, which might attenuate the preventive effect of sufficient insulin secretion and provision of AA during the post-exercise recovery period, as mentioned above. This view is similar for the timing of nutrient intake after RE. Rasmussen et al. (2000) reported that essential AA and CHO intake one hour after RE tended to suppress MPB during the post-exercise recovery period compared to the effect of nutritional intake 3 h after RE [8]. Therefore, it is speculated that a sufficient nutrient intake as soon as possible after RE may be more effective than that before RE, at least for the suppression of MPB.

According to the findings of Bird et al., the combined intake of essential AA and CHO during acute RE suppressed 3-MH concentration (i.e., MPB response) more than either AA, CHO, or placebo intake alone [3]. Importantly, this transient suppression of MPB resulted in the greatest muscle anabolic response following resistant training: RE with combined essential AA and CHO intake throughout a twelve-week training period most effectively increased LBM among the test conditions [3]. This finding suggests that a chronic muscle hypertrophy response may be expected by examining the acute MPB response. In this regard, we investigated the effect of the timing of nutrient intake on acute RE by focusing on acute MPB response by 3-MH concentration. Although changes in 3-MH are believed reflective of skeletal MPB, the precise contribution of myofibrillar proteins such as myosin and actin to overall MPB following resistance exercise remains to be elucidated [2,33]. Indeed, the local level of interstitial 3-MH secreted in the skeletal muscle was affected by exercise intensity and modality [34]. To clarify the effect of the timing of nutrient intake on RE-induced positive muscle protein net balance, long-term training intervention experiments may be needed.

Again, negative muscle protein net balance is brought about by a long period of fasting after the last meal in the morning [21]. In this connection, a habitual substantial mixed meal in the morning is associated with maintenance of muscle mass [22]. Considering our present finding, the optimal timing of RE in the morning would be before such a meal to achieve further positive muscle protein net balance. Importantly, it is feasible to apply such nutrient timing to other meals (i.e., not only breakfast but also lunch and dinner). Therefore, whether consuming meals immediately after RE at any of the three meals of the day can promote anabolic response should be further elucidated to develop an effective nutritional prescription for maintaining and increasing muscle mass.

There are some limitations in this study. First, the timing of meal intake before RE was 1.5 h before the start of RE. In this study, the reason for the long period before RE was to prevent indigestion of the ingested food as a result of exercising [35] because, unlike a supplement, participants ingested a meal that may take a long time to digest. Second, protein provision was standardized as absolute amount, not relative to individual body volume. In this study, 0.88 ± 0.02 g protein/kg body mass was provided in the mixed meal, which is far above the “breakpoint” for the anabolic stimulation ensured for muscle hypertrophy (i.e., 0.24 g protein/kg body mass) [17,36], and hence enough to see the nutrient effect. Indeed, we showed similar changes in the plasma 3-MH and 3-MH/LBM levels. Overall, our crossover-design study might dampen confounding factors derived from individual differences. Third, MPS was not assessed in this study. Further studies are necessary to develop an effective nutritional prescription in terms of timing for maintaining and increasing muscle mass.

## 5. Conclusions

In summary, for the first time, the present study investigated the acute MPB response to a substantial mixed meal after RE, before RE, or RE without nutrient intake in a crossover-design study. As a result, meal intake after RE was found to suppress MPB (3-MH) to a greater extent than meal intake before RE and RE without meal intake. We conclude that the intake of nutrient- and protein-dense whole foods after RE may improve anabolic response to a greater extent than meal intake before RE or without a meal by suppressing MPB in the morning.
